# Bakuchiol kills *Staphylococcus aureus* persisters and potentiates colistin activity against *Acinetobacter baumannii* persisters

**DOI:** 10.3389/fphar.2025.1592183

**Published:** 2025-05-15

**Authors:** Kayeong Suh, Yeju Lee, Seongeun Baek, Jin Kim, Jinbeom Seo, Yung-Hun Yang, Jae-Seok Kim, Wonsik Lee, Eun Kyoung Seo, Wooseong Kim

**Affiliations:** ^1^ College of Pharmacy, Graduate School of Pharmaceutical Sciences, Ewha Womans University, Seoul, Republic of Korea; ^2^ School of Pharmacy, Sungkyunkwan University, Suwon, Republic of Korea; ^3^ Advanced Materials Program, Department of Biological Engineering, College of Engineering, Konkuk University, Seoul, Republic of Korea; ^4^ Department of Laboratory Medicine, Kangdong Sacred Heart Hospital, Hallym University College of Medicine, Seoul, Republic of Korea

**Keywords:** persisters, antibiotic resistance, natural products, MRSA, *Acinetobacter* *baumannii*

## Abstract

Infections associated with bacterial persisters are challenging to cure because they can evade antibiotics and regrow, often resulting in relapse. Current antibiotics are not optimized to target persisters, highlighting the urgent need for new therapeutics. Here, we report that bakuchiol, a plant-derived natural product, exhibits anti-persister and adjuvant properties. Bakuchiol eradicates persisters formed by the gram-positive bacterium *Staphylococcus aureus* at 8 μg/mL and, in combination with 1 μg/mL colistin, completely eliminates persisters formed by the gram-negative bacterium *Acinetobacter baumannii*. Mechanistic analyses revealed that bakuchiol selectively disrupted bacterial membrane phospholipids while sparing mammalian membranes and exhibited low cytotoxicity. In *Acinetobacter baumannii* persisters, bakuchiol likely damages phospholipid patches in the outer membrane, causing nominal lethality but facilitating membrane permeabilization. This activity synergizes with colistin, which targets the lipooligosaccharide layer, resulting in the mutual reinforcement of their bactericidal effects. These findings highlight the potential of dual glycolipid–phospholipid targeting as a strategy to combat gram-negative persisters and highlight natural products as valuable sources for anti-persister therapeutics with membrane selectivity.

## 1 Introduction

Bacteria can evade antibiotic attack by altering their metabolic or growth states. This trait, known as antibiotic tolerance, is distinct from genetically acquired antibiotic resistance. Unlike resistance, antibiotic tolerance is a nonheritable, transient defense mechanism that enables bacteria to survive high concentrations of lethal antibiotics (Gollan et al., 2019; Balaban et al., 2019). Most antibiotics function by inhibiting essential biosynthetic processes, such as cell wall and nucleic acid synthesis, which are active during bacterial growth (Darby et al., 2023). Furthermore, the cellular uptake of certain antibiotics, such as aminoglycosides, largely depends on the proton motive force, which is maintained during cellular respiration and active bacterial growth (Davis, 1987; Allison et al., 2011). When bacteria enter reduced metabolic or dormant states, antibiotic targets become unavailable or antibiotic uptake is blocked. This allows them to survive antibiotic treatment, resulting in the formation of bacterial persisters. Although not yet fully understood, stringent and SOS responses, triggered by environmental stresses, such as nutrient limitations and toxic chemicals, play an important role in persister formation (Gerdes and Maisonneuve, 2012; Dörr et al., 2010; Völzing and Brynildsen, 2015; Wu et al., 2012; Rowe et al., 2020). Clinically, bacterial persisters are often found in biofilms and inside host cells, including macrophages, which contribute to the failure of antibiotic therapy (Niu et al., 2024). Current antibiotic arsenals are largely ineffective against bacterial persisters, highlighting the unmet need for new antimicrobial therapeutics targeting persisters.

Membrane-active agents have a valuable potential as therapeutic agents against bacterial persisters because membrane integrity is crucial for bacterial viability, regardless of the metabolic or growth state (Hurdle et al., 2011; Kim et al., 2018a). These agents offer several advantages, such as fast killing kinetics, a low likelihood of resistance development, and the ability to potentiate other antimicrobials (Hurdle et al., 2011; Kim et al., 2018b; Kim et al., 2018c). However, conventional membrane-active agents have significant drawbacks, particularly their reduced bactericidal activity against persisters. For instance, the Food and Drug Administration-approved antibiotics daptomycin and colistin exhibit diminished bactericidal activity against persister cells (Urbaniec et al., 2023; Heo et al., 2024). Similarly, some membrane-active small molecules, such as bitin-S, adarotene, and PQ401, rapidly permeabilize the membranes of actively growing methicillin-resistant *Staphylococcus aureus* (MRSA) but show reduced potency against MRSA persister cells (Kim et al., 2019; Radlinski et al., 2019; Kim et al., 2020). Furthermore, membrane-active agents often lack selectivity between bacterial and mammalian membranes (Hurdle et al., 2011). Addressing these challenges is essential for the development of effective membrane-active therapeutics to combat bacterial persisters.

Plant-derived natural products are considered valuable reservoirs for antimicrobial development because of their structural diversity and various modes of action (Porras et al., 2021). Alkaloids, phenolic derivatives, and terpenoids have been reported to exhibit antimicrobial activity (Woo et al., 2023). Their antibacterial mechanisms include inhibiting DNA and cell wall synthesis (Wu et al., 2008; Karkare et al., 2013), suppressing efflux pumps (Kuete et al., 2010), and impairing the cytoplasmic membrane (Nourbakhsh et al., 2022; Kumar and Engle, 2023). Interestingly, certain flavonoids, a type of phenolic plant-derived natural product, have been shown to selectively disrupt bacterial membranes while protecting erythrocyte membranes (Wang et al., 2019; Veiko et al., 2023). Considering these unique properties, we hypothesized that particular plant-derived natural products may exhibit bactericidal activity against bacterial persisters without causing significant damage to mammalian membranes.

In this study, through screening of plant-derived natural compounds, we identified the bactericidal effects of bakuchiol, a monoterpene derived from the leguminous plant *Psoralea corylifolia*, on MRSA persisters. Bakuchiol kills MRSA persisters by selectively disrupting membrane phospholipid bilayers, leading to the leakage of essential cellular components, such as adenosine triphosphate (ATP) and proteins. Notably, although this compound alone is ineffective against persisters formed by the gram-negative bacterium *Acinetobacter baumannii*, its combination with colistin shows an enhanced lethal effect against *A. baumannii* persister cells. The anti-persister and adjuvant properties of bakuchiol, along with its selectivity for bacterial phospholipid bilayers, highlight the potential of plant-derived natural products for treating antibiotic-tolerant bacterial persisters.

## 2 Materials and methods

### 2.1 Bacterial strains and growth conditions

The *S. aureus* strains MW2 BAA-1707 (Baba et al., 2002), ATCC 33591, and ATCC 43300; clinical MRSA isolates (HLSA 16278, 17064, 17078, 18380, 18807, 18840, 18883, 18888, 20835, and 21008) (Heo et al., 2024); and vancomycin-resistant *S. aureus* (VRSA) strain VRS1 (Weigel et al., 2003) were grown in tryptic soy broth (TSB) (BD, Franklin Lakes, NJ, United States). *Enterococcus faecium* E007 (Garsin et al., 2001), *Enterococcus faecalis* MMH594 (Shankar et al., 1999), and vancomycin-resistant *E. faecium* (VRE) strains C68 (Carias et al., 1998), WB312 (Thorisdottir et al., 1994), and WC196 (Thorisdottir et al., 1994) were cultured in brain–heart infusion (BHI) broth (BD, Franklin Lakes, NJ, United States). *Klebsiella pneumoniae* ATCC 77326, *A. baumannii* ATCC 17978, *A. baumannii* ATCC 19606, *lpxC*-deletion mutant of *A. baumannii* ATCC 19606 (Moison et al., 2017), *Pseudomonas aeruginosa* PA14 (Rahme et al., 1995), and *Enterobacter aerogenes* ATCC 13048 were grown in Luria Bertani (LB) broth (BD, Franklin Lakes, NJ, United States). All bacterial strains were incubated overnight at 37°C with shaking at 250 rpm.

### 2.2 Preparation of antimicrobial and compound stocks

Oxacillin (Sigma-Aldrich, Cat# PHR2488), gentamicin (Sigma-Aldrich, Cat# G1264), vancomycin (Sigma-Aldrich, Cat# V2002), daptomycin (Tocris, Cat# 3917), colistin (Sigma-Aldrich, Cat# C4461), and benzyldimethylhexadecylammonium chloride (16-BAC, Sigma-Aldrich, Cat# B4136) were dissolved in deionized sterile water. Ciprofloxacin (Sigma-Aldrich, Cat# 17850) was dissolved in 0.1 N HCl. Bakuchiol (Sigma-Aldrich, Cat# SMB00604) and linezolid (Tocris, Cat# 3765) were dissolved in dimethyl sulfoxide (DMSO). For experiments involving daptomycin, calcium chloride (CaCl_2_) was added at a final concentration of 50 μg/mL. The antimicrobial stock solutions were prepared at concentrations of 5 or 10 mg/mL. For each experimental procedure, aliquots of the stock solutions were diluted to the desired concentrations in the appropriate medium or buffer.

### 2.3 Preparation of bacterial persisters

*Staphylococcus aureus* MW2 and *S. aureus* VRS1 persister cells were generated using a stationary-phase induction method (Kim et al., 2019). To prepare stationary-phase persisters, an overnight culture of *S. aureus* was diluted to 25 mL of TSB in a 250-mL flask at a ratio of 1:10,000 and incubated at 37°C with shaking at 250 rpm for 24 h. After reaching the stationary phase, the cells were washed three times with phosphate-buffered saline (PBS) and resuspended in PBS at a concentration of approximately 10^8^ colony-forming units (CFU)/mL. The antibiotic tolerance of the persister cells was evaluated by counting the number of CFUs after a 4-h treatment with 100× minimum inhibitory concentration (MIC) of vancomycin, gentamicin, or ciprofloxacin for *S. aureus* MW2 and 100× MIC of daptomycin or linezolid for *S. aureus* VRS1.

*Acinetobacter baumannii* ATCC 19606 persister cells were generated using a ciprofloxacin induction method (Dörr et al., 2010). An overnight culture of *A. baumannii* ATCC 19606 was diluted to 25 mL of LB in a 250-mL flask at a ratio of 1:10,000 and incubated at 37°C with shaking at 250 rpm for 24 h. The culture was then treated with 100× MIC of ciprofloxacin for 4 h. To confirm antibiotic tolerance, these cells were subjected to an additional 4-h challenge with 100× MIC of ciprofloxacin, meropenem, or gentamicin, which resulted in no decrease in viability.

### 2.4 Posttreatment regrowth screening assays

An in-house collection of 102 plant-derived natural compounds was used to screen for anti-persister agents. Each compound was dissolved in DMSO at a stock concentration of 10 mg/mL and diluted into media or buffer to a final concentration of 64 μg/mL for screening. Screening was performed separately on exponentially growing MRSA cells and persisters. To prepare exponential-phase *S. aureus* MW2 cells, an overnight culture was diluted into 25 mL of fresh TSB in a 250-mL flask at a ratio of 1:10,000 and incubated at 37°C with shaking at 250 rpm for approximately 3 h until reaching the mid-log phase (OD_600_ ∼0.2). The optical density (OD_600_) was measured using an Eppendorf BioSpectrometer Basic (Eppendorf, Germany). Persister cells were prepared as described in Section 2.3.

For the screen using growing cells, 100 µL of exponential-phase *S. aureus* MW2 in TSB was added to prewarmed 96-well assay plates (Corning Falcon, Cat# 353072, United States), which contained 100 µL of each natural product in TSB at a 64 μg/mL. For the persister screen, 100 µL of *S. aureus* MW2 persisters in PBS was added to prewarmed 96-well assay plates, which contained 100 µL of each natural product in PBS at a final concentration of 64 μg/mL. The plates were incubated at 37°C for 4 h with constant shaking at 450 rpm using a Titramax 1,000 shaker (Heidolph, Germany). After incubation, 2 µL of each well was transferred into 198 µL of fresh cation-adjusted Muller-Hinton (CaMH) medium in new 96-well plates, which were incubated at 37°C for 18 h, and OD_600_ was measured using a Cytation 5 multimode reader (BioTek, United States). Z-scores were calculated by subtracting the average OD_600_ of all wells from the OD_600_ of each individual well, then dividing by the standard deviation (SD) of the OD_600_ values across all wells.

### 2.5 MIC determination

The MICs of the antimicrobials were determined using the microbroth dilution method, following the Clinical and Laboratory Standards Institute guidelines (Institute, 2012). The test agents were serially diluted twofold in CaMH or BHI broth to achieve concentrations ranging from 128 to 0.125 μg/mL in 96-well assay plates (Corning Falcon, Cat# 353072, United States). Overnight bacterial cultures, except for *Enterococcus* spp., were diluted to a final concentration of 1 × 10^6^ CFU/mL in CaMH broth. For *Enterococcus* spp., the cultures were diluted in BHI broth. Fifty microliters of the diluted bacterial suspension was added to an equal volume of the test compound in the assay plates. The negative control comprised untreated bacterial cultures. The assay plates were incubated overnight at 37°C, and OD_600_ was measured using a Cytation 5 multimode reader (BioTek, United States). Growth inhibition was defined as an OD_600_ < 0.1. Each MIC was determined in three independent experiments.

### 2.6 Persister-killing assay

The persister cells of *S. aureus* MW2, *S. aureus* VRS1, or *A. baumannii* ATCC 19606 were prepared as described in Section 2.3. A 500-µL aliquot of persister cells suspended in PBS at a concentration of approximately 10^8^ CFU/mL was added to each well of a 96-well deep plate (Bioneer, Cat# 9006), along with an equal volume of PBS containing twice the desired concentrations of each tested agent. The mixtures were incubated in a Titramax 1,000 shaker (Heidolph, Germany) at 37°C with shaking at 450 rpm. Samples were collected at hourly intervals, serially diluted 10-fold in PBS, and spot-plated onto CaMH agar plates. Colonies were counted after overnight incubation at 37°C. As previously described (Doern, 2014), synergy was defined as a ≥2-log reduction in CFU/mL by the combination treatment compared to the most active single agent alone after 4 h. All experiments were performed in biological triplicate.

### 2.7 Mammalian cell culture

The immortalized human embryonic kidney cell line HEK-293 and the human lung carcinoma cell line A549 were maintained in Dulbecco’s Modified Eagle Medium (DMEM) (Gibco, Cat# 11965-092) containing 15-mM HEPES, 10% fetal bovine serum, and penicillin–streptomycin (100 units/mL), and the cells were incubated in a humidified 5% CO_2_ incubator at 37°C. Once the cultures reached 70%–80% confluence, the cells were transferred to tissue culture-treated 96-well plates, with each well containing 100 μL of the culture medium.

### 2.8 Membrane permeabilization assay

Changes in bacterial membrane permeability were assessed using the membrane-impermeable DNA-binding dye SYTOX Green, as previously described (Kim et al., 2018b). Bacterial cells were washed three times with PBS and adjusted to an OD_600_ of 0.5. SYTOX Green was added to the washed cells at a final concentration of 5 μM, and the samples were incubated at 37°C in the dark for 30 min. A 50-μL aliquot of each sample was transferred to a black 96-well plate (Greiner Bio-One, Cat# 655090), with each well containing the desired concentration of the tested compounds. The fluorescence was measured at room temperature using a Cytation 5 multimode plate reader (BioTek) with excitation and emission wavelengths of 485 and 525 nm, respectively. All experiments were performed in triplicate.

### 2.9 Evaluation of intracellular ATP leakage

Intracellular ATP leakage was assessed using the RealTime-Glo™ Extracellular ATP Assay (Promega, Madison, WI, United States), as previously described (Heo et al., 2024). For bacterial persisters, the cells were washed three times with PBS and resuspended to an OD_600_ of 0.5. A 50-μL aliquot of the suspension was added to each well of a black, clear-bottom 96-well plate (Greiner Bio-One, Cat# 655090) pre-filled with 50 μL of the tested compounds at twice the desired final concentration. The plate was incubated at 37°C with shaking at 450 rpm for 1 h using a Titramax 1,000 shaker (Heidolph, Germany). For mammalian cells, HEK-293 or A549 cells grown to a confluence of approximately 80% in black, clear-bottom 96-well plates were washed twice with PBS and once with pure DMEM. The cells were then exposed to varying concentrations of bakuchiol or 16-BAC in serum- and antibiotic-free DMEM, with a final volume of 100 μL/well, and incubated in a humidified 5% CO_2_ atmosphere at 37°C for 1 h. In both assays, after incubation, 33.4 μL of the 4× ATP assay reagent mixture was added to each well, and luminescence was measured using a Cytation 5 multimode plate reader (BioTek). All experiments were performed in triplicate.

### 2.10 Evaluation of protein leakage

Intracellular protein leakage from bacterial persisters was evaluated using the Micro BCA™ Protein Assay Kit (Thermo-Fisher Scientific, Cat# 23235, United States) with slight modifications to the manufacturer’s protocol. Persister cells were washed three times with PBS and resuspended to an OD_600_ of approximately 0.5. A 500-μL aliquot of the suspension was added to each microcentrifuge tube containing 500 μL of the tested compounds at twice the desired final concentration. The tubes were incubated statically at 37°C for 1 h, followed by centrifugation at 14,000 rpm for 3 min. A 500-μL aliquot of the supernatant from each tube was transferred to a new set of microcentrifuge tubes containing 500 μL of the working reagent. After thorough mixing, the tubes were incubated at 60°C in a water bath for 1 h and allowed to cool to room temperature. Subsequently, 100 μL of each tube was transferred to a 96-well plate, and absorbance at 562 nm was measured using a Cytation 5 multimode plate reader (BioTek, United States). All experiments were performed in triplicate.

### 2.11 Membrane lipid binding assay

The binding affinity of bakuchiol was evaluated based on changes in the MIC as previously described (Heo et al., 2024). Phospholipids including phosphatidylglycerol (PG) (Avanti Polar Lipids, Cat# 841188P), lysyl phosphatidylglycerol (Lysyl-PG) (Avanti Polar Lipids, Cat# 840520P), and cardiolipin (CL) (Avanti Polar Lipids, Cat# 841199P) were dissolved in methanol to prepare 10-mg/mL stock solutions. Lipopolysaccharides (LPS) from *Escherichia coli* O111:B4 (Sigma-Aldrich, Cat# L2630) were dissolved in deionized sterile water. In 96-well plates, a twofold serial dilution of bakuchiol and each lipid was prepared. Bakuchiol was diluted along the x-axis, and the lipids were diluted along the y-axis, starting at a concentration of 64 μg/mL. To each well containing 50 μL of the bakuchiol–lipid mixture, 50 μL of bacterial suspension at 1 × 10^6^ CFU/mL was added, resulting in a final volume of 100 μL. The plates were incubated overnight at 37°C, and OD_600_ was measured using a Cytation 5 multimode reader (BioTek). Growth inhibition was defined as OD_600_ < 0.1. All experiments were performed in triplicate with three biological replicates.

### 2.12 Hemolysis assay

Washed 25% human red blood cells (RBCs) were obtained from Innovative Research (Novi, MI, United States) and diluted to a final concentration of 4% in PBS. A 100-μL aliquot of the diluted RBCs was added to 100 μL of twofold serial dilutions of bakuchiol in PBS or 2% Triton X-100 (positive control) in a 96-well plate, with the starting concentration of bakuchiol at 64 μg/mL. The plate was incubated at 37°C for 1 h and then centrifuged at 500 *g* for 5 min. A 50-μL aliquot of the supernatant from each well was carefully transferred to a new 96-well plate, and absorbance was measured at 540 nm. Hemolysis (as a percentage) was calculated using the following formula: (A_540_ of compound-treated sample − A_540_ of nontreated sample)/(A_540_ of 1% Triton X-100-treated sample − A_540_ of nontreated sample) × 100. The experiments were independently replicated three times.

### 2.13 Cytotoxicity assay

HEK-293 or A549 cells grown to a confluence of approximately 80% in 96-well plates were washed twice with PBS and once with pure DMEM. The cells were then exposed to varying concentrations of bakuchiol or 16-BAC in serum- and antibiotic-free DMEM, with a final volume of 100 μL per well, and incubated in a humidified 5% CO_2_ atmosphere at 37°C for 24 h. During the final hour of incubation, 10 μL of WST-1 reagent (Sigma-Aldrich, Cat# 5015944001) was added to each well. WST-1 reduction, which indicates cell viability, was measured at 450 nm using a Cytation 5 multimode plate reader (BioTek). Cell viability was calculated as a percentage relative to nontreated control wells. All experiments were performed in triplicate.

## 3 Results

### 3.1 A regrowth screen identified bakuchiol as effective against MRSA persisters

**FIGURE 1:**
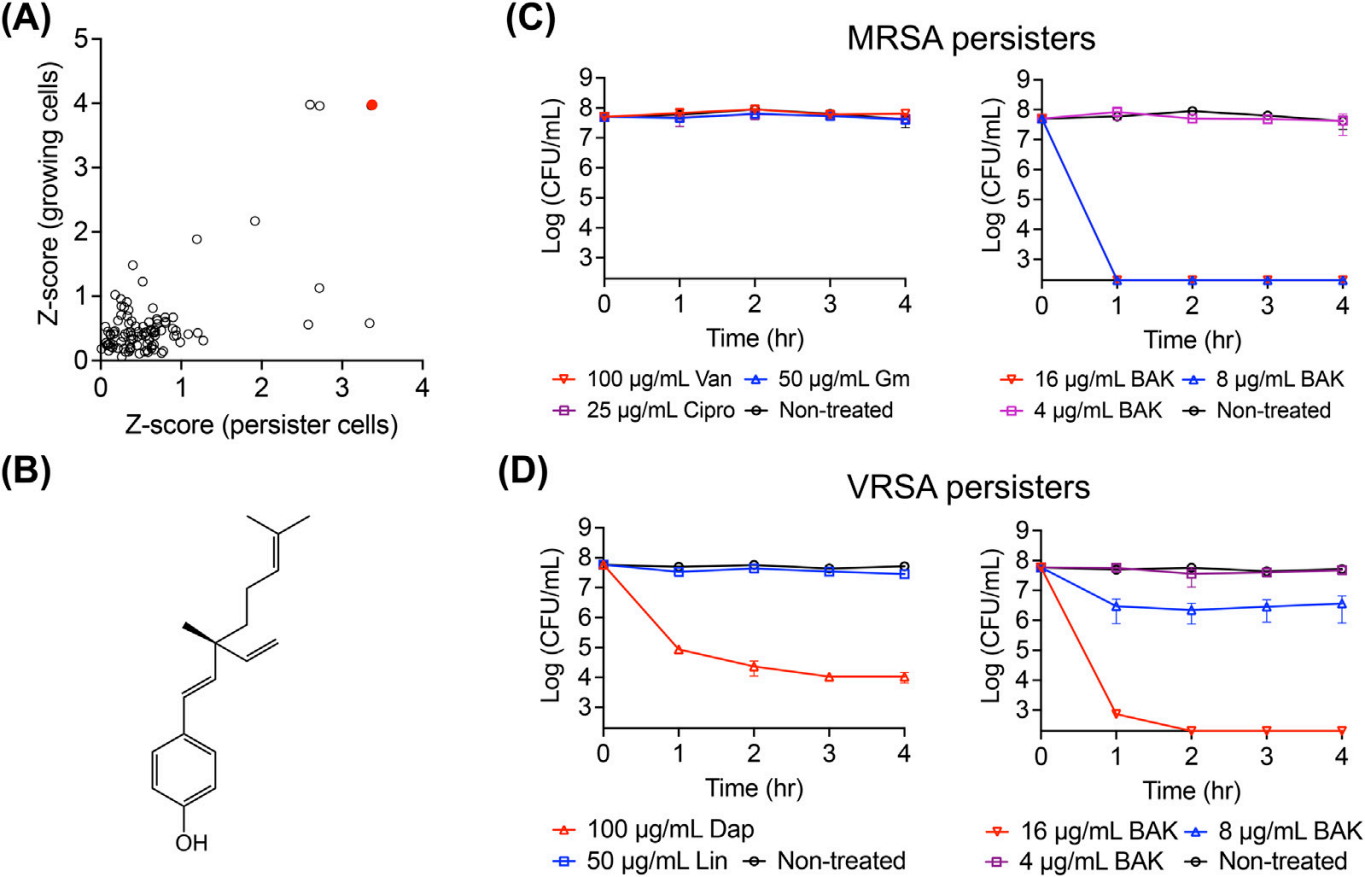
Identification of the persister-killing activity of bakuchiol. **(A)** Screening results from a natural product library. Actively growing MRSA MW2 cells and MRSA MW2 persister cells were treated with 64 μg/mL of each natural product for 4 h. Each treated sample was then inoculated into fresh CaMH and incubated for 18 h, and the optical density at 600 nm (OD_600_) was measured. Z-scores for regrowth of treated-growing and treated-persister cells were calculated by obtaining the OD_600_ value of each sample, subtracting the mean OD_600_ of all tested samples, and dividing by the standard deviation (SD) of the OD_600_ values from all tested samples. **(B)** Chemical structure of bakuchiol. **(C,D)** Viability of MRSA MW2 **(C)** and VRSA VRS1 **(D)** persister cells treated with 100× MIC of conventional antibiotics—vancomycin (Van), gentamicin (Gm), ciprofloxacin (Cipro), daptomycin (Dap), and linezolid (Lin)—or the indicated concentrations of bakuchiol (BAK) for 4 h. Data points at the x-axis detection limit represent a CFU count of 2 × 10^2^ CFU/mL. Individual data points (n = 3 biologically independent samples) are shown, with error bars representing the mean ± SD.

We aimed to identify natural products that are effective against both actively growing MRSA and MRSA persisters. To achieve this, we screened an in-house library of 102 plant-derived natural products using posttreatment regrowth assays, in which growing MRSA cells and MRSA persister cells were treated with 64 μg/mL of each natural compound for 4 h. After treatment, the cells were diluted to fresh CaMH media at a ratio of 1:100 and incubated at 37°C for 18 h, during which regrowth will not occur if the natural products killed the cells. The killing potency was assessed by measuring the optical density and was quantitatively ranked based on the Z-score values. Bakuchiol emerged as the top hit, showing the highest Z-score values for both growing and persister cell treatments (Figures 1A,B). Although bakuchiol was previously known for its antimicrobial activity (Katsura et al., 2001), its anti-persister activity has not been reported.

To confirm the antimicrobial activity of bakuchiol, its MIC was first determined. Consistently, this natural compound exhibited MIC values of 4–8 μg/mL against gram-positive pathogenic bacteria, such as *S. aureus* MW2, *E. faecium* E007, and *E. faecalis* MMH594 (Table 1). However, bakuchiol exhibited no antimicrobial activity against gram-negative bacteria, such as *K. pneumoniae*, *A. baumannii*, *P. aeruginosa*, and *E. aerogenes*, at concentrations up to 64 μg/mL (Table 1), consistent with a previous report showing no activity of native bakuchiol against multiple Gram-negative species (Li et al., 2021). Notably, bakuchiol showed potent antimicrobial activity, with MIC values of 4–8 μg/mL, against a panel of multidrug-resistant *S. aureus* and *E. faecium* strains, including clinical MRSA isolates, VRSA, and VRE strains (Table 2).

**TABLE 1:** Antimicrobial activity of bakuchiol against major human pathogens.

Strains	Bakuchiol	Vancomycin	Gentamicin	Ciprofloxacin
*Staphylococcus aureus* MW2	8	1	0.5	0.25
*Enterococcus faecium* E007	8	0.5	>64	64
*Enterococcus faecalis* MMH594	4	1	>64	0.125
*Klebsiella pneumoniae* ATCC 77326	>64	>64	0.5	0.063
*Acinetobacter baumannii* ATCC 17978	>64	>64	0.5	0.125
*Pseudomonas aeruginosa* PA14	>64	>64	2	0.063
*Enterobacter aerogenes* ATCC 13048	>64	>64	0.25	0.063

**TABLE 2:** Antimicrobial activity of bakuchiol against multidrug-resistant gram-positive pathogens.

Bacterial strains	Bakuchiol	Oxacillin	Vancomycin	Daptomycin
*Staphylococcus aureus* strains				
ATCC 33591	4	>64	1	0.5
ATCC 43300	4	32	0.5	0.25
HLSA 16278	4	>64	1	1
HLSA 17064	4	>64	0.5	1
HLSA 17078	4	64	0.5	0.5
HLSA 18380	4	64	0.5	1
HLSA 18807	4	>64	0.5	0.5
HLSA 18840	8	>64	1	1
HLSA 18883	4	>64	0.5	1
HLSA 18888	4	>64	0.5	0.5
HLSA 20835	8	>64	1	1
HLSA 21008	8	64	1	1
VRS1	4	>64	>64	1
*Enterococcus faecium* strains				
C68	4	>64	32	16
WB312	4	>64	4	16
WC196	4	>64	16	16

Next, the anti-persister activity of bakuchiol was confirmed using the stationary-phase persister cells of the MRSA strain MW2 and the VRSA strain VRS1 (Kim et al., 2019). Consistent with previous reports, stationary-phase MRSA MW2 cells exhibited no decrease in viability after 4-h exposure to 100× MIC of vancomycin, gentamicin, or ciprofloxacin, which is characteristic of the high antibiotic tolerance of persister cells (Figure 1C). However, bakuchiol at 8 μg/mL (1× MIC) eradicated approximately 5 × 10^7^ CFU/mL MRSA persister cells within 1 h (Figure 1C). Similarly, although daptomycin at 100× MIC failed to eliminate VRSA persisters, 16 μg/mL bakuchiol completely killed approximately 5 × 10^7^ CFU/mL VRSA persisters within 2 h (Figure 1D). These results show that bakuchiol possesses bactericidal activity against highly antibiotic-tolerant persister cells formed by antibiotic-resistant *S. aureus*.

### 3.2 Bakuchiol kills MRSA persisters through severe membrane disruption

**FIGURE 2:**
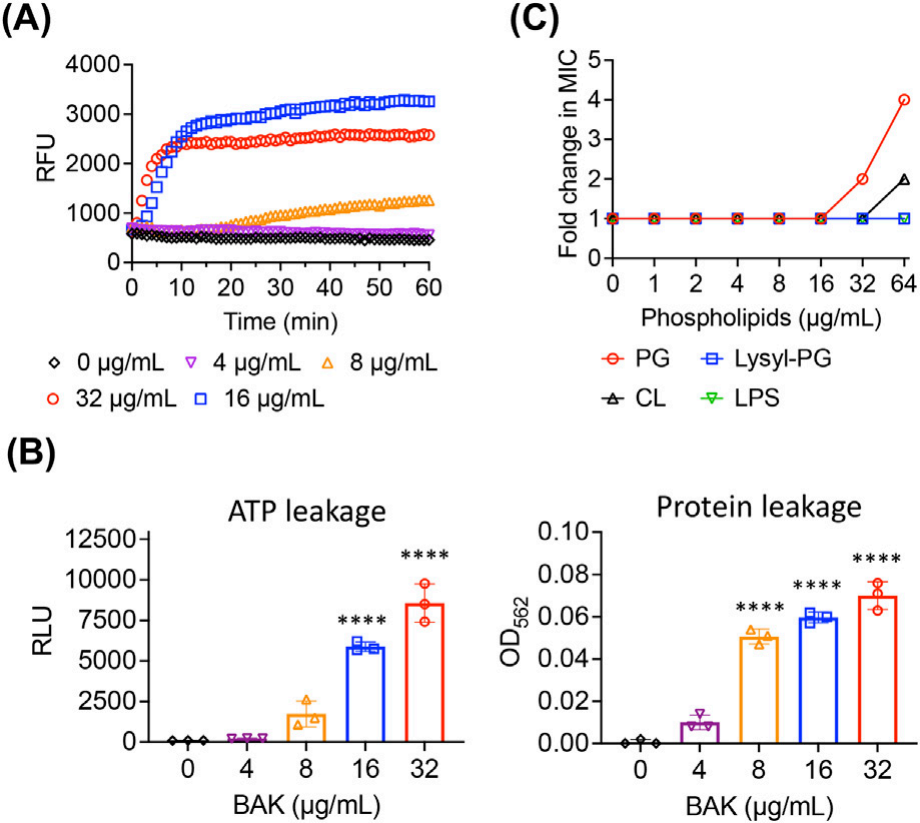
Bakuchiol disrupts the MRSA persister membrane. **(A)** Uptake of SYTOX Green (Ex = 485 nm, Em = 525 nm) by MRSA MW2 persister cells treated with the indicated concentrations of bakuchiol (BAK). Results are presented as means (n = 3 biologically independent samples); error bars are omitted for clarity. RFU indicates relative fluorescence units. **(B)** Leakage of cellular ATP and proteins from MRSA MW2 persister cells treated with various BAK concentrations for 1 h. Leakage was measured using an ATP luminescence assay kit and a Micro BCA protein assay kit. Individual data points are shown; error bars represent means ± SD (n = 3). Statistical differences were analyzed using one-way analysis of variance with the *post hoc* Tukey test (****p < 0.0001). **(C)** Changes in BAK MIC against MRSA MW2 in the presence of phosphatidylglycerol (PG), lysyl phosphatidylglycerol (Lysyl-PG), cardiolipin (CL), or lipopolysaccharides (LPS) were evaluated using checkerboard microdilution assays. The lipid component concentrations ranged from 0 to 64 μg/mL. The experiment was performed in triplicate, with all replicates showing consistent MIC changes.

The mechanisms by which bakuchiol kills MRSA persisters were investigated. Because bacterial membranes are among the most effective targets for killing persisters (Kim et al., 2018a; Hurdle and Deshpande, 2018), the effects of bakuchiol on MRSA persister membranes were specifically examined. Bakuchiol rapidly induced SYTOX Green membrane permeabilization in MRSA persisters (Figure 2A). To further evaluate the severity of membrane disruption, leakage of intracellular components was assessed. As shown in Figure 2B, bakuchiol promoted the dose-dependent leakage of intracellular ATP and proteins from MRSA persisters.

To further investigate the interaction between bakuchiol and membrane lipid bilayers, we assessed its binding affinity to key *S. aureus* membrane lipid components, including PG, Lysyl-PG, and CL. If bakuchiol specifically binds to any of these lipid components, their external addition could interfere with its interaction with bacterial membranes, resulting in reduced antimicrobial activity. Although supplementation with Lysyl-PG did not affect the MIC, the addition of PG and CL at 64 μg/mL increased the MIC of bakuchiol by up to 4 and 2 folds, respectively (Figure 2C). These findings suggest that the antimicrobial activity of bakuchiol against *S. aureus* arises from its ability to interact with membrane lipid bilayers, with a selective affinity for specific phospholipids, such as PG and CL.

### 3.3 Bakuchiol exhibits low membrane activity and cytotoxicity in mammalian cells

**FIGURE 3:**
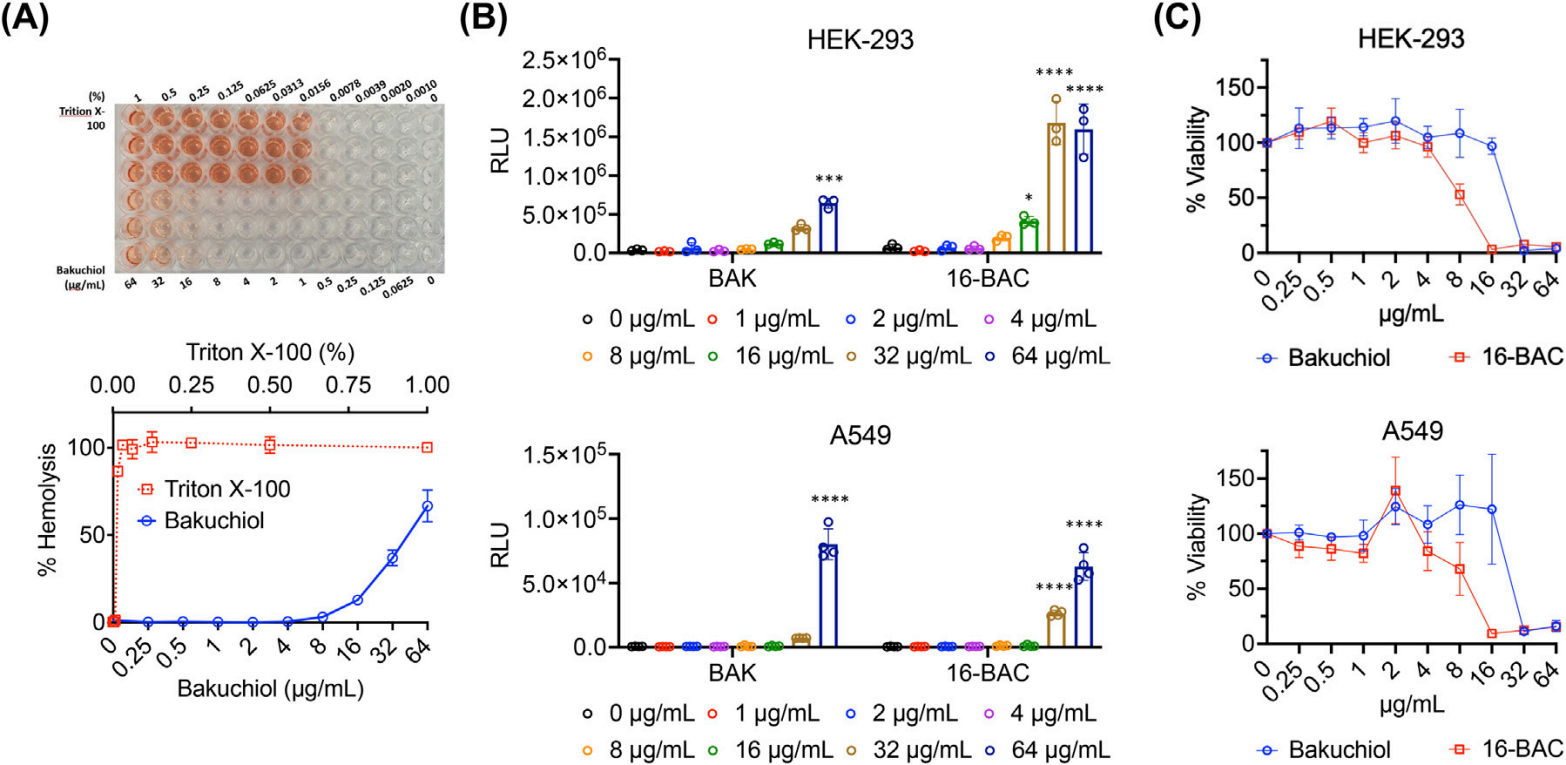
Bakuchiol exhibits relatively low membrane activity and cytotoxicity in mammalian cells. **(A)** Human red blood cells (2%) were treated with various BAK concentrations for 1 h at 37°C. A sample treated with 1% Triton X-100, which induces 100% hemolysis, was used as the positive control. Results are presented as means ± SD (n = 3). **(B)** ATP leakage from HEK-293 or A549 cells after treatment with the indicated concentrations of BAK for 1 h was evaluated using an ATP luminescence assay kit. The cationic detergent 16-BAC was used as the positive control. Individual data points are shown; error bars denote means ± SD (n = 3). Statistical differences were analyzed using two-way analysis of variance and the *post hoc* Tukey test (*p < 0.05, ***p < 0.001 and ****p < 0.0001). **(C)** The viability of HEK-293 and A549 cells was assessed after treatment with various BAK and 16-BAC concentrations for 24 h. Cell viability was determined based on the conversion of WST-1 dye to formazan by viable cells, measured at 450 nm. Results are presented as means ± SD (n = 3).

Next, the effects of bakuchiol on mammalian membranes were assessed. Bakuchiol exhibited a median hemolytic concentration (HC_50_) of 44 μg/mL and caused nominal hemolysis up to 16 μg/mL (Figure 3A), a concentration at which it effectively kills antibiotic-resistant *S. aureus* persister cells (Figures 1C,D). Similarly, bakuchiol did not induce significant intracellular ATP leakage from either the human embryonic kidney cell line HEK-293 or the human lung adenocarcinoma cell line A549 up to 32 μg/mL (Figure 3B). Furthermore, cytotoxicity in HEK-293 and A549 cells was evaluated 24 h after treatment, and we found that bakuchiol had median lethal concentrations (LC_50_) of 22 and 30 μg/mL, respectively (Figure 3C). Importantly, no detectable cell death was observed in either HEK-293 or A549 cells at concentrations up to 16 μg/mL (Figure 3C). These findings indicate that bakuchiol exhibits high selectivity for bacterial membranes over mammalian membranes and demonstrates nominal cytotoxicity at concentrations effective for persister cell killing.

### 3.4 Glycolipids in the outer membrane inhibit the membrane activity of bakuchiol

The mechanisms by which bakuchiol is ineffective against gram-negative bacteria were investigated. In contrast to gram-positive bacteria, gram-negative bacteria possess an additional outer membrane, where the outer leaflet comprises glycolipids, such as LPS or lipooligosaccharide (LOS), and the inner leaflet comprises phospholipids. Because the antimicrobial activity of bakuchiol is associated with its ability to disrupt membrane integrity, we hypothesized that glycolipids in gram-negative bacteria may nullify its membrane activity. To test this, whether bakuchiol has a binding affinity for LPS was first assessed. As shown in Figure 2C, the external addition of LPS at concentrations up to 64 μg/mL did not alter the MIC of bakuchiol against MRSA MW2, indicating that bakuchiol is unlikely to interact with LPS.

**FIGURE 4:**
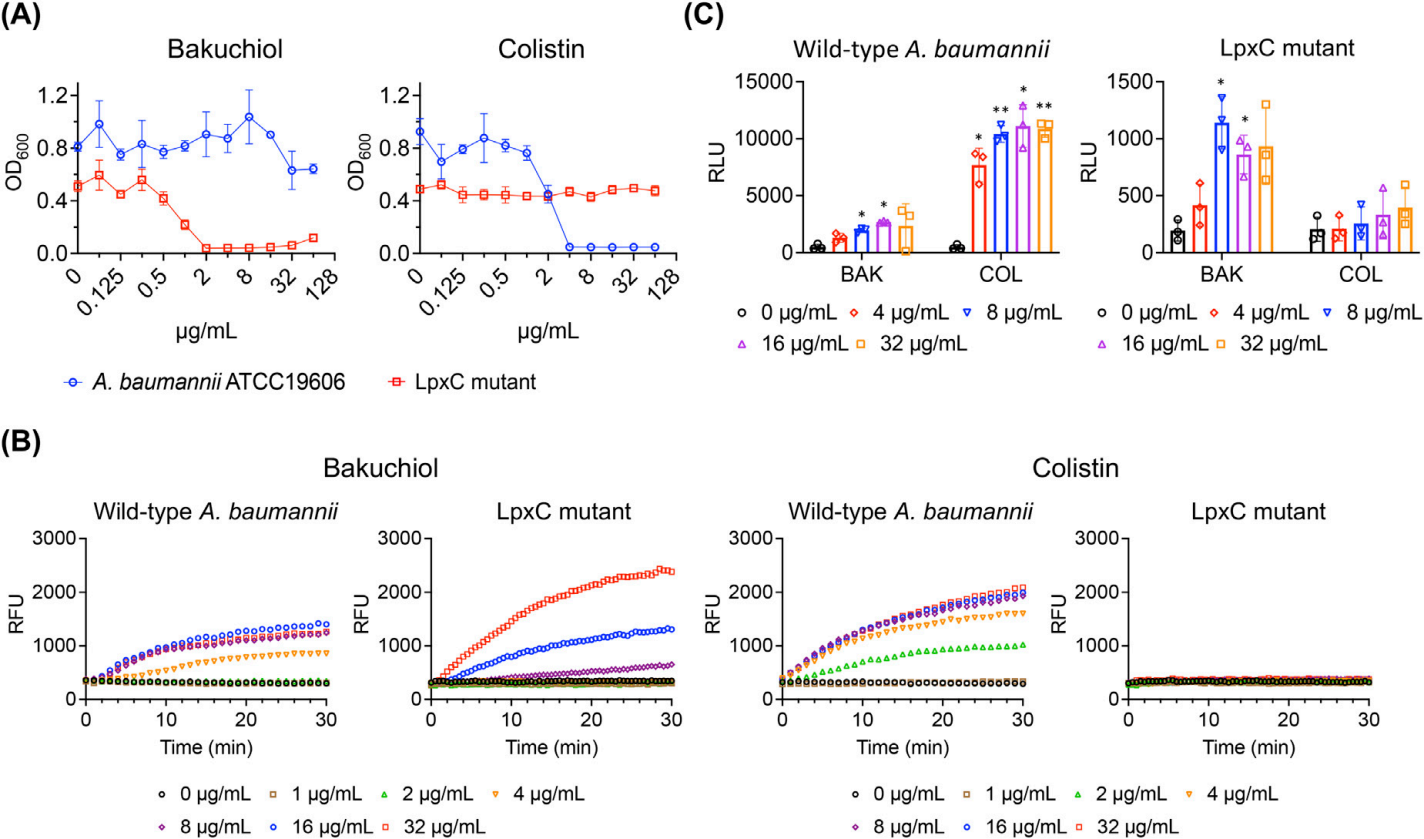
Bakuchiol induces membrane permeabilization but does not inhibit growth or cause ATP leakage in *Acinetobacter baumannii* due to the presence of LOS. **(A)** Growth of wild-type *Acinetobacter baumannii* ATCC 19606 and its lipooligosaccharide-deficient mutant (LpxC mutant) treated with varying concentrations of bakuchiol or colistin for 18 h. Bacterial growth was assessed by measuring the optical density at 600 nm (OD_600_). Data are presented as means ± SD (n = 3). **(B)** Membrane permeabilization assessed by SYTOX Green uptake (Ex = 485 nm, Em = 525 nm) in wild-type *Acinetobacter baumannii* ATCC 19606 and LpxC mutant treated with the indicated concentrations of bakuchiol or colistin. Data are presented as means (n = 3 biologically independent samples); error bars are omitted for clarity. RFU indicates relative fluorescence units. **(C)** Intracellular ATP leakage from wild-type *Acinetobacter baumannii* and the LpxC mutant treated with varying concentrations of bakuchiol (BAK) or colistin (COL) for 1 h, measured using an ATP luminescence assay kit. Individual data points are shown, with error bars representing means ± SD (n = 3). Statistical significance was analyzed using two-way analysis of variance with Tukey’s *post hoc* test (*p < 0.05, **p < 0.01).

We reasoned that in the absence of glycolipids in the outer membrane, bakuchiol may exhibit antimicrobial activity against gram-negative bacteria. To test this, an *lpxC*-deleted mutant strain of *A. baumannii* ATCC 19606, referred to as the LpxC mutant, was used (Moffatt et al., 2010). LpxC is a key enzyme in the synthesis of lipid A, the lipid anchor of LOS; thus, the LpxC mutant lacks LOS in its outer membrane. Notably, *A. baumannii* is among the few gram-negative bacteria that can grow without the LOS or LPS component in their outer membrane (Moffatt et al., 2010; Powers and Trent, 2018). We compared the antimicrobial activity of bakuchiol between wild-type *A. baumannii* ATCC 19606, which has an intact outer membrane, and the LpxC mutant. Although bakuchiol failed to inhibit the growth of *A. baumannii* ATCC 19606 at concentrations up to 64 μg/mL, it completely prevented the growth of the LpxC mutant at concentrations of ≥2 μg/mL (Figure 4A). This growth inhibition pattern was distinct from that of colistin, which targets the lipid A of LOS and inhibits the growth of *A. baumannii* ATCC 19606 but not the LpxC mutant (Figure 4A). These results indicate that bakuchiol can disrupt the phospholipid bilayers but not the LPS or LOS layers of the outer membranes, explaining its lack of antimicrobial activity against gram-negative bacteria.

### 3.5 Bakuchiol permeabilizes the *Acinetobacter baumannii* membrane with reduced damage severity

Next, the effects of bakuchiol on the *A. baumannii* membrane was evaluated. As expected, bakuchiol caused membrane permeabilization in the LpxC mutant (Figure 4B), which was susceptible to bakuchiol with an MIC of 2 μg/mL (Figure 4A). Interestingly, despite its lack of antimicrobial activity (MIC >64 μg/mL), bakuchiol induced membrane permeabilization in *A. baumannii* ATCC 19606 (Figure 4B). In contrast, colistin induced membrane permeabilization in *A. baumannii* ATCC 19606 but not in the LpxC mutant (Figure 4B). This is consistent with its antimicrobial activity, as colistin exhibited an MIC of 4 μg/mL against *A. baumannii* ATCC 19606 and an MIC of >64 μg/mL against the LpxC mutant. Unlike its effect on membrane permeability, bakuchiol did not significantly cause ATP leakage from *A. baumannii* ATCC 19606, although it caused ATP leakage from the LpxC mutant (Figure 4C). In contrast, colistin induced ATP leakage from *A. baumannii* ATCC 19606 but not from the LpxC mutant (Figure 4C). These observations suggest that bakuchiol induces sufficient membrane damage in *A. baumannii* to increase membrane permeability; however, this membrane damage was not severe enough to cause growth inhibition.

### 3.6 Bakuchiol and colistin exhibit synergistic killing activity against *Acinetobacter baumannii* persisters

**FIGURE 5:**
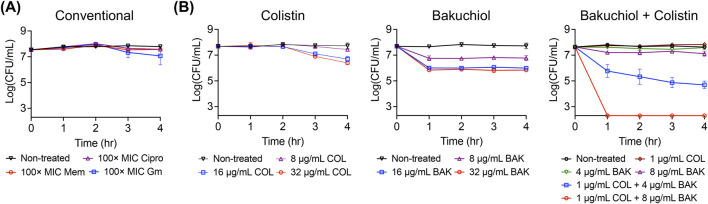
Bakuchiol and colistin exhibit synergistic bactericidal activity against *Acinetobacter baumannii* persisters. **(A)** *Acinetobacter baumannii* persisters were treated with 100× MIC ciprofloxacin (Cipro), meropenem (Mem), or gentamicin (Gm) for 4 h, and the titer of viable cells was determined. No decrease in viability confirms the antibiotic tolerance of *Acinetobacter baumannii* persisters. **(B)** *Acinetobacter baumannii* persister cells were challenged with the indicated concentrations of bakuchiol, colistin, or their combination. Data points at the x-axis detection limit represent a CFU count of 2 × 10^2^ CFU/mL. Individual data points (n = 3 biologically independent samples) are shown, with error bars representing means ± SD.

Gram-negative bacterial persisters are more challenging to eliminate than their gram-positive counterparts because of the presence of double membranes. Bakuchiol disrupts phospholipid bilayers and permeabilizes outer membranes and colistin specifically targets lipid A; therefore, we hypothesized that their combination may effectively kill gram-negative persisters. To test this hypothesis, we first generated *A. baumannii* ATCC 19606 persister cells by treating overnight cultures with a high concentration of ciprofloxacin (Dörr et al., 2010). As shown in Figure 5A, the resulting *A. baumannii* persister cells exhibited no decrease in viability after 4-h treatment with 100× MIC ciprofloxacin, meropenem, or gentamicin, confirming their tolerance to antibiotics with different modes of action.

Next, the killing potency of bakuchiol, colistin, and their combination was evaluated. Bakuchiol alone at 32 μg/mL caused an approximately 2-log reduction in persister viability after 4 h, whereas colistin alone at 32 μg/mL caused an approximately 1-log reduction over the same period (Figure 5B). Notably, although either 8 μg/mL bakuchiol or 1 μg/mL colistin individually caused nominal killing, their combination completely eradicated the persister cells within 1 h (Figure 5B). Moreover, 4 μg/mL bakuchiol combined with 1 μg/mL colistin resulted in more than a 3-log reduction in viability, whereas neither agent alone exhibited significant activity at these concentrations (Figure 5B). In a time-kill assay, synergism is defined as a 2-log or greater reduction in CFU/mL achieved by the combination compared with the most active individual agent (Doern, 2014). Accordingly, our results demonstrate that bakuchiol and colistin exhibited synergistic killing potency against *A. baumannii* persisters.

### 3.7 Bakuchiol restores colistin’s membrane disruption ability against *Acinetobacter baumannii* persisters

**FIGURE 6:**
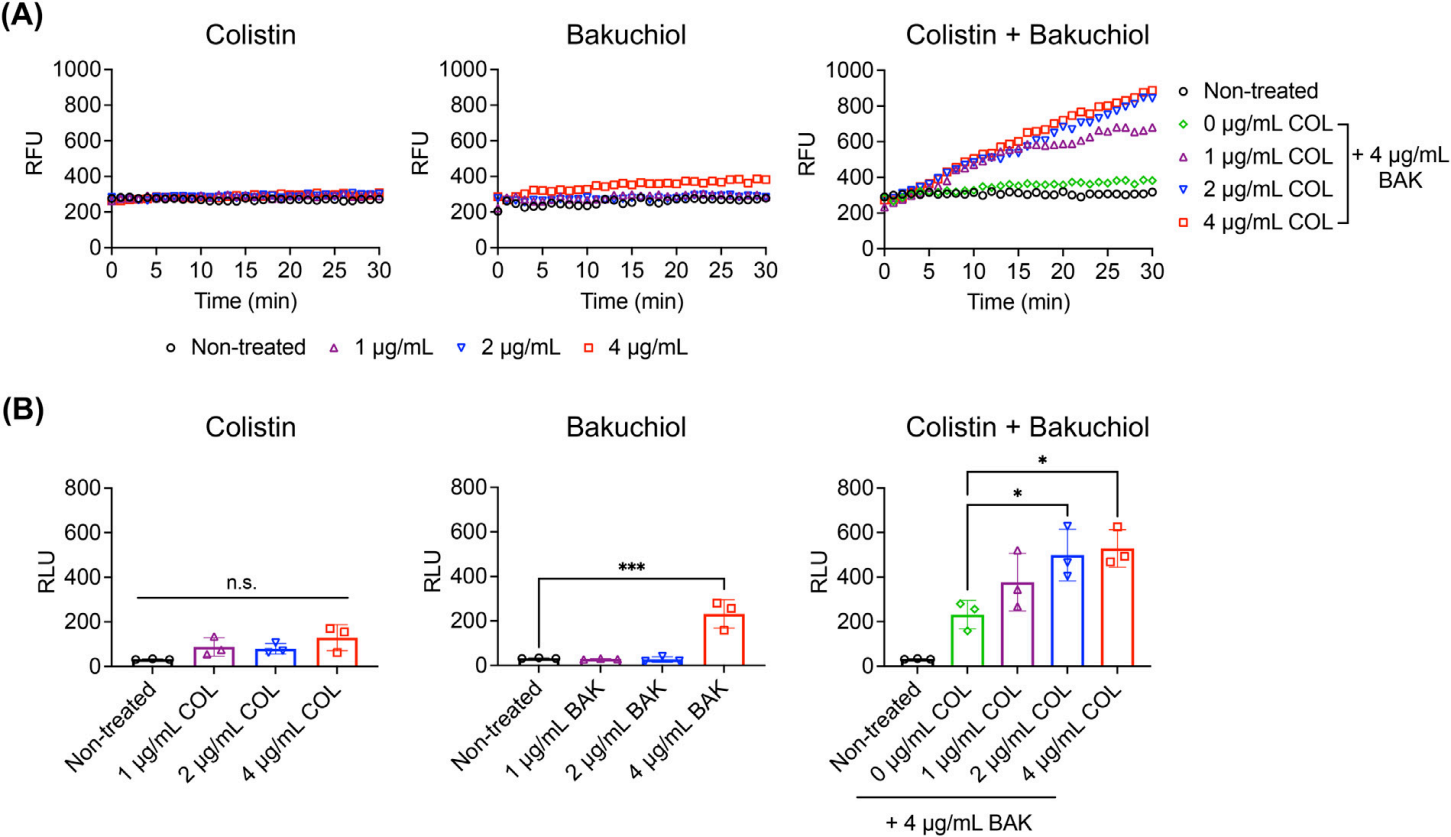
Bakuchiol restores the membrane-disrupting activity of colistin against *Acinetobacter baumannii* persisters. **(A)** Uptake of SYTOX Green (Ex = 485 nm, Em = 525 nm) by *Acinetobacter baumannii* ATCC 19606 persister cells treated with colistin (COL), bakuchiol (BAK), or their combination at the indicated concentrations. Results are representative of three independent experiments. RFU denotes relative fluorescence units. **(B)** Intracellular ATP leakage from *Acinetobacter baumannii* persisters treated with COL, BAK, or their combination for 1 h at the indicated concentrations, measured using an ATP luminescence assay kit. Individual data points are shown, and error bars represent means ± SD (n = 3). RLU denotes relative luminescence units. Statistical significance was analyzed using one-way analysis of variance with Tukey’s *post hoc* test (*p < 0.05, ***p < 0.001).

The mechanism underlying the synergism between bakuchiol and colistin against *A. baumannii* persisters was explored. Consistent with its reduced killing potency, colistin at concentrations up to 4 μg/mL failed to cause either membrane permeabilization or intracellular ATP leakage in *A. baumannii* persisters (Figures 6A,B). In contrast, treatment with bakuchiol at 4 μg/mL not only induced membrane permeabilization but also promoted ATP leakage from persister cells (Figures 6A,B). Notably, when combined with 4 μg/mL bakuchiol, colistin restored its ability to induce membrane permeabilization and ATP leakage (Figures 6A,B). These findings demonstrate that bakuchiol reinstates colistin’s membrane activity against *A. baumannii* persisters, highlighting enhanced membrane disruption as a key mechanism underlying their synergistic bactericidal effects.

## 4 Discussion

Infections associated with bacterial persisters often relapse because of the survival and regrowth of persister cells. These infections are difficult to treat because current antibiotics are not designed to eliminate dormant cells. The development of innovative agents that specifically target persisters is crucial for overcoming this medical challenge. Although membrane-targeting agents are promising anti-persister therapeutics, their development is limited by drawbacks such as low selectivity between bacterial and mammalian membranes and reduced bactericidal activity against persisters. We reasoned that natural products may contain membrane-active agents that can overcome these limitations. Among them, we identified the plant-derived compound bakuchiol, which eradicates the persisters formed by the gram-positive bacterium MRSA at 8 μg/mL within 1 h. Furthermore, at the same concentration, in combination with 1 μg/mL colistin, bakuchiol completely eliminated the persisters formed by the gram-negative bacterium *A. baumannii*. Notably, at 8 μg/mL, bakuchiol did not exhibit detectable damage to mammalian membranes or cytotoxicity. The anti-persister and adjuvant properties of bakuchiol support the potential of natural products as therapeutic leads against bacterial persisters.

Bakuchiol has previously been shown to exhibit antibacterial activity against *S. aureus* through inhibition of DNA gyrase (Tripathi et al., 2024). However, this mechanism is unlikely to account for its activity against persisters, as these cells exist in a non-replicating, metabolically quiescent state in which DNA replication is largely inactive. Indeed, antibiotics such as ciprofloxacin—which target DNA gyrase and topoisomerase IV (Hooper and Jacoby, 2016)—fail to reduce persister viability even at 100× MIC (Figure 1C). In contrast, our results support a membrane-targeting mechanism for bakuchiol’s anti-persister activity, as demonstrated by increased SYTOX Green uptake and leakage of intracellular ATP and proteins—hallmarks of membrane disruption.

Several studies have reported that not all membrane-targeting antimicrobials possess effective bactericidal activity against *S. aureus* persisters (Heo et al., 2024; Radlinski et al., 2019; Kim et al., 2020). Although the mechanisms underlying the diminished killing activity on persisters are not fully understood, alterations in the lipid composition and physical properties of the persister membranes are believed to contribute to this reduction in bactericidal activity (Zou et al., 2024). For instance, the membrane-acting antibiotic daptomycin exhibits significantly reduced potency against *S. aureus* stationary-phase persisters, which is likely due to the conversion of phosphatidylglycerol, the primary binding target of daptomycin, into CL, a phospholipid that inhibits the membrane-disrupting activity of daptomycin (Taylor and Palmer, 2016; Zhang et al., 2014; Jiang et al., 2019). In contrast to daptomycin, our findings indicate that bakuchiol binds to both PG and CL (Figure 2C). This dual binding affinity may explain its consistent killing activity against *S. aureus* persisters even in the presence of lipid composition changes that typically impair the effectiveness of other membrane-targeting antibiotics.

The effects of bakuchiol on *A. baumannii* are intriguing. Although bakuchiol does not inhibit the growth of *A. baumannii* at concentrations up to 64 μg/mL (Table 1; Figure 4A), it kills approximately 99% of the persister cells within 4 h at 16 μg/mL (Figure 5B). The discrepancy in antimicrobial activity between the two growth states may be due to the change in the proportion of glycolipids and phospholipids in the membranes. The outer membrane of gram-negative bacteria is asymmetric, with an outer leaflet composed primarily of LPS or LOS and an inner leaflet composed of phospholipids. However, phospholipids can become mislocalized to the outer leaflet, forming patches of phospholipids (Jia et al., 2004; Guest and Silhavy, 2023). To maintain membrane asymmetry, *A. baumannii* employs retrograde transport systems to relocate mislocalized phospholipids from the outer leaflet to the inner membrane and enzymatic machinery to degrade these misplaced phospholipids (Powers and Trent, 2018; Powers et al., 2020). Notably, retrograde phospholipid transport requires ATP hydrolysis. Furthermore, glycolipids, such as LOS and LPS, are synthesized in the cytoplasm and inner membrane and then transported to the outer membrane through ATP-dependent processes (Lundstedt et al., 2021).

We propose that in actively growing *A. baumannii*, this machinery efficiently removes phospholipid patches from the outer leaflet and fills these gaps with newly synthesized and transported LOS. This process likely reduces the likelihood of bakuchiol targeting phospholipids. In contrast, during the persister state, the retrograde transport system and LOS synthesis/transport may be inactive or are operating at reduced capacity, increasing the presence of phospholipid patches in the outer leaflet (Nikaido, 2003; Nilsson et al., 2020). This expanded exposure of phospholipids could provide bakuchiol with readily accessible targets, enabling it to effectively disrupt the outer membrane of *A. baumannii* persisters. We further propose that the reduced bactericidal potency and membrane activity of colistin against *A. baumannii* persisters (Figures 5B, 6A,B) may result from the altered membrane composition. In particular, an increase in phospholipid proportions and a decrease in LOS—colistin’s primary target—may occur due to the inactivation of phospholipid patch removal and LOS synthesis in persister cells.

Although neither bakuchiol nor colistin alone is effective, their combination induces severe damage sufficient to eradicate *A. baumannii* persisters. This synergism may arise from mutual reinforcement of their membrane-disrupting activities. Colistin targets and disrupts LOS-rich domains in the outer membrane. In persister cells in which LOS synthesis may be inactive, damaged LOS domains may be replaced by phospholipids translocated from the inner leaflet, increasing the proportion of phospholipid patches in the outer membrane. Bakuchiol subsequently interacts with these phospholipid patches and the inner phospholipid membrane, causing irreparable damage that ultimately leads to persister cell death. These findings suggest that the combination of agents targeting glycolipids and phospholipids provides an effective strategy to combat gram-negative persisters, warranting further investigation to validate this approach.

In conclusion, we demonstrated that the plant-derived natural product bakuchiol acts as a bactericidal agent against the persisters formed by the gram-positive bacterium *S. aureus* and as a potentiator for colistin against the persisters formed by the gram-negative bacterium *A. baumannii*. Bakuchiol exhibits membrane selectivity that favors bacterial over mammalian membranes and shows low cytotoxicity. Our findings highlight the potential of natural products as promising resources for the discovery and development of anti-persister therapeutics. Furthermore, the synergistic mechanism between membrane-active agents targeting distinct membrane components offers valuable insights for the development of strategies to address persistent, hard-to-treat infections caused by gram-negative pathogens.

## Data Availability

The original contributions presented in the study are included in the article/supplementary material, further inquiries can be directed to the corresponding authors.
